# Multicenter Evaluation of the Cepheid Xpert GBS LB XC Test

**DOI:** 10.1128/jcm.01356-22

**Published:** 2022-12-06

**Authors:** Phyu M. Thwe, Matthew L. Faron, David T. Pride, Amorina Cruz, Derek Gerstbrein, Karen A. Nahmod, Lindsay Bigham, Xiaotang Du, Xixi Lu, Stephanie Moya, Ping Ren

**Affiliations:** a Department of Pathology, University of Texas Medical Branch, Galveston, Texas, USA; b Department of Pathology, Montefiore Medical Center, Bronx, New York, USA; c Department of Pathology, The Medical College of Wisconsin, Milwaukee, Wisconsin, USA; d Department of Pathology, University of California, San Diego, La Jolla, California, USA; e Department of Medicine, University of California, San Diego, La Jolla, California, USA; f Cepheid, Sunnyvale, California, USA; Mayo Clinic

**Keywords:** group B *Streptococcus* (GBS), Xpert GBS LB XC test, sensitivity, specificity

## Abstract

Early-onset neonatal sepsis due to Streptococcus agalactiae (group B Streptococcus [GBS]) infection is one of the leading causes of newborn mortality and morbidity. The latest guidelines published in 2019 recommended universal screening of GBS colonization among all pregnant women and intrapartum antibiotic prophylaxis for positive GBS. The updated procedures allow rapid molecular-based GBS screening using nutrient broth-enriched rectovaginal samples. Commercially available molecular assays for GBS diagnosis target mainly the *cfb* gene, which encodes a hemolysin protein responsible for producing the Christie-Atkins-Munch-Petersen (CAMP) factor. *cfb* is considered a conserved gene in essentially all GBS isolates. However, false-negative GBS results on Cepheid Xpert GBS and GBS LB tests due to deletions in or near the region that encodes *cfb* were reported recently. Therefore, the new Xpert GBS LB XC test was developed. This study is a multicenter evaluation of the new test for GBS identification from nutrient broth-enriched rectal/vaginal samples from antepartum women. A total of 621 samples were prospectively enrolled. The samples were tested with the Xpert GBS LB XC test, the composite comparator method, which included the Hologic Panther Fusion GBS test combined with bacterial culture, followed by matrix-assisted laser desorption ionization–time of flight mass spectrometry (MALDI-TOF MS) identification, and bacterial culture alone, followed by MALDI-TOF MS identification. The respective sensitivity and specificity of the Xpert GBS LB XC test were 99.3% and 98.7% compared to the composite comparator method and 99.1% and 91.8% compared to bacterial culture alone with MALDI-TOF MS identification. Overall, the Xpert GBS LB XC test performed comparatively to the composite comparator method and is equivalent to traditional bacterial culture followed by MALDI-TOF MS.

## INTRODUCTION

Streptococcus agalactiae, i.e., group B Streptococcus (GBS), colonizes the gastrointestinal and genitourinary tracts of healthy women, with a colonization rate of 15% to 30% in pregnant women in the United States (1, 2). Although GBS rarely causes severe infections in immunocompetent people, it is a leading cause of neonatal sepsis and meningitis in newborns (2). The implementation of universal antepartum GBS screening guidelines by the Centers for Disease Control and Prevention (CDC) in 2010 resulted in a decreased rate of early-onset neonatal sepsis (EOS) from 1.5 per 1,000 births in the 1980s to 0.34 per 1,000 births in 2016 (1, 3, 4). In 2019, clinical and diagnostic guidelines for the prevention of neonatal GBS infection were published by the American College of Obstetricians and Gynecology (ACOG), American Society for Microbiology (ASM), and American Association of Pediatrics (AAP) and replaced the 2010 CDC guidelines (1, 4, 5). The latest guidelines recommend antepartum screening of GBS colonization at 36 0/7 to 37 6/7 weeks of gestation (1, 4). Both nucleic acid amplification tests (NAATs) and conventional GBS cultures from enriched broth medium for GBS screening are recommended testing methods. However, direct testing of the specimens without enrichment is not endorsed due to reduced sensitivity (5).

Several NAATs are commercially available and target a variety of conserved regions of the GBS genome (3). Our study presents a multicenter evaluation of the Cepheid Xpert GBS LB XC test, which was recently cleared by the Food and Drug Administration (FDA). The Xpert GBS LB XC test is a real-time PCR test for the detection of GBS from vaginal/rectal swabs enriched in nutrient broth. The Xpert GBS LB XC test is a modified version of the currently available Xpert GBS LB test. The Xpert GBS LB test targets the conserved region within the CAMP factor (*cfb*) gene (3), which encodes the Christie-Atkins-Munch-Petersen (CAMP) factor in GBS. The CAMP factor, also known as protein B, enhances the hemolysin activity of Staphylococcus aureus (6). The CAMP test, in addition to other biochemical tests, is traditionally used to confirm the identification of GBS in clinical microbiology laboratories (7). The *cfb* gene is considered present in almost all GBS strains; therefore, most molecular assays target different conserved regions of *cfb* to detect GBS. A recent study by Tickler et al. demonstrated the presence of four distinct chromosomal deletions in the *cfb* gene, resulting in false-negative Xpert GBS LB results (8). These mutant strains represented less than 1% and 7% of the total isolates tested from surveillance samples collected from the clinical laboratories at three distinct geographic sites in the United States and one in Ireland, respectively (8). Most mutant isolates were recovered from a single participating site (8). The overall prevalence of these chromosomal deletions is low; however, the finding that the majority of mutant strains originated from a single geographical region, contributing to 7.1% of the total samples tested (8), causes concern about the diagnostic test accuracy as the organism continues to evolve.

The newly developed Xpert GBS LB XC test targets unique sequences in two GBS chromosomal regions: a coding region for glucosyl transferase family protein (GTFP) and a region in the *LysR* family transcriptional regulator of GBS. We compared the performance of the Xpert GBS LB XC test with a composite comparator method and with the gold-standard bacterial culture method followed by MALDI-TOF MS identification. The composite comparator method was the combination of (i) the Hologic Panther Fusion GBS assay, which targets the conserved regions in the *cfb* and SIP genes, and (ii) conventional bacterial culture isolation after broth enrichment, followed by matrix-assisted laser desorption ionization–time of flight mass spectrometry (MALDI-TOF MS) identification. Overall, the Xpert GBS LB XC test performed comparatively to the composite comparator method and is equivalent to traditional cultures.

## MATERIALS AND METHODS

### Specimen collection and enrichment.

Dual vaginal/rectal swab specimens (*n* = 621) were collected from pregnant women at antepartum as part of the standard of care (SOC) GBS testing using a Cepheid collection device (part number 900-0370) or a swab in nonnutritive transport medium. Per ASM guidelines for the detection and identification of GBS, optimal recovery of viable organisms from swabs is achieved when the swab is preserved using nonnutritive transport medium (5). Either the Cepheid collection device or a swab in nonnutritive transport medium used at the study sites per their SOC GBS testing would have met the ASM guidelines. Specimens were inoculated in Lim broth per institutional policies and incubated for 18 to 24 h at 35 to 37°C in ambient air or 5% CO_2_. Eligible leftover samples were enrolled in this study from February to March 2021 at the University of Texas Medical Branch, the Medical College of Wisconsin, and the University of California, San Diego.

### Sample storage and processing.

After enrichment, SOC testing was conducted per the sites’ institutional policies and procedures. Residual eligible enriched Lim broth samples were stored at 2 to 8°C for ≤72 h prior to the Xpert GBS LB XC test on-site. Aliquots of the residual Lim broth specimens were shipped overnight with freezer packs to the reference laboratory for enriched bacterial culture, within 48 h of the completion of enrichment incubation. Meanwhile, another aliquot was added to the Hologic transport medium according to the manufacturer’s instructions and shipped with ice packs within 1 day of addition to the Hologic transport medium tube, for the Panther Fusion GBS assay to be performed within 5 days of enrichment. All sites received the institutional review board (IRB) approval through the Advarra IRB services prior to collecting and testing leftover samples in this study.

### Testing by enriched bacterial culture.

Colonies subcultured on tryptic soy with sheep blood agar (SBA) and Columbia nalidixic acid (CNA) agar from the LB-enriched samples were phenotypically characterized as either GBS (large gray to white glistening colonies with a narrow zone of β-hemolysis) or atypical (large gray to white glistening colonies with no hemolysis resembling enterococci). Presumptive GBS colonies and atypical colonies were subcultured on an SBA plate and tested using MALDI-TOF MS (Bruker Daltonics, Billerica, MA; software/library, RUO MBT Compass v4.1.100.10). If GBS was present in a study sample, it was reported as Streptococcus agalactiae. Enriched bacterial culture testing and identification via MALDI-TOF MS were performed at International Health Management Associates, Inc. (Schaumburg, IL).

### Instruments.

The Xpert GBS LB XC (Cepheid, Sunnyvale, CA) and the Panther Fusion GBS (Hologic, Inc., San Diego, CA) tests are real-time PCR *in vitro* diagnostic tests for the qualitative detection of GBS from enriched vaginal/rectal swab specimens collected from pregnant women at antepartum. The Xpert GBS LB XC test, performed on GeneXpert instrument systems, provides results in less than 1 h and includes an early assay termination (EAT) function that enables early result reporting for positive results. GeneXpert instrument systems automate and integrate sample purification, nucleic acid amplification, and detection of target sequences. The systems require single-use disposable cartridges that contain the PCR reagents and host the PCR process. Similarly, the Panther Fusion GBS assay is performed on the Panther Fusion system, which automates specimen processing, including cell lysis, nucleic acid capture, amplification, and target detection. However, the turnaround time for the Panther Fusion GBS assay is more prolonged, about 2.5 h, but with a large capacity as one of its advantages. At the time of this study, the Hologic Panther Fusion GBS assay was FDA cleared, and the Xpert GBS LB XC test has since received FDA clearance.

### Assay procedures.

Following enrollment, the LB-enriched samples were stored at 2 to 8°C for ≤72 h prior to Xpert GBS LB XC testing. Following the instruction for use (IFU) provided by Cepheid, Xpert GBS LB XC testing was performed by dipping a clean swab into the enriched Lim broth and then transferring the swab to the sample chamber of the test cartridge for testing. For Panther Fusion GBS testing, a 1-mL aliquot of the Lim broth sample was added to the Aptima transport medium according to the manufacturer’s instructions and transferred to the testing site. Panther Fusion GBS testing was initiated within 5 days of the LB-enriched samples being added to the Aptima transport medium, per the manufacturer’s instructions. A separate aliquot of the Lim Broth sample was sent to the reference laboratory for enriched bacterial culture and identification.

### Comparison to composite comparator method.

The sensitivity and specificity of the Xpert GBS LB XC test were estimated relative to a composite comparator method based on the algorithm in Table 1. For the composite comparator, a sample was considered positive if either the enriched bacterial culture or the Panther Fusion test were positive; a sample was considered negative when both the enriched bacterial culture and the Panther Fusion test were negative (Table 1). If the Xpert results did not agree with the enriched bacterial culture and/or Panther Fusion GBS results, no additional testing was performed. All eligible specimens enrolled in this study had valid culture results.

**TABLE 1:** Composite comparator method

Panther Fusion GBS test result	Enriched bacterial culture result	
	Positive	Negative
Positive	+	+
Negative	+	−
No result	+	Excluded

### Statistical analysis.

Statistical analyses were performed using SAS v9.4 (SAS Institute, Cary, NC).

## RESULTS

### Sample accountability.

A total of 627 samples were enrolled in this study from all three study sites. Five samples were excluded due to protocol deviations. In these five samples, four swab specimens were stored at room temperature for over 24 h prior to inoculation. One sample was not tested using the Xpert GBS LB XC due to a cartridge supply issue. In addition, one sample was excluded from the study due to an indeterminate result after repeat Xpert GBS LB XC testing. Of the 622 samples tested with the Xpert GBS LB XC test in this study, 9 yielded indeterminate results on the initial test. These 9 samples were retested, and 8 returned valid results. The initial indeterminate rate was 1.4% (9/622), and the final indeterminate rate was 0.2% (1/622). A total of 621 samples were included in the final data analysis.

### Demographics.

The study participants belonged to the following age groups: 14 to 17 years (*n* = 10), 18 to 24 years (*n* = 117), 25 to 34 years (*n* = 357), and ≥35 years (*n* = 137). The age distribution for specimens included in the final data set is presented in Table 2.

**TABLE 2:** Age distribution of participants

Age (yrs)	No. of participants	%
14–17	10	1.6
18–24	117	18.8
25–34	357	57.5
≥35	137	22.1
Total	621	100.0

### Performance of the Xpert GBS LB XC.

The performance of the Xpert GBS LB XC test compared to the composite comparator method according to the algorithm shown in Table 1 is presented in Table 3. The sensitivity, specificity, positive predictive value (PPV), negative predictive value (NPV), and positivity rate with 95% confidence intervals (CIs) are provided.

**TABLE 3:** Performance of the Xpert GBS LB XC test versus the composite comparator method

Xpert GBS LB XC result & test performance	Composite comparator results		
	Positive	Negative	Total
Positive	142	6	148
Negative	1	472	473
Total	143	478	621
Sensitivity	99.3%	95% CI^*a*^ = 96.1%−99.9%	
Specificity	98.7%	95% CI = 97.3%−99.4%	
Positive predictive value	95.9%	95% CI = 91.4%−98.1%	
Negative predictive value	99.8%	95% CI = 98.8%−100.0%	
Positivity rate	23.0%	95% CI = 19.9%−26.5%	

a CI, confidence interval.

As shown in Table 3, the sensitivity and specificity of the Xpert GBS LB XC test compared to the composite comparator method were 99.3% and 98.7%, respectively. The sensitivity and specificity of the Xpert GBS LB XC test compared to enriched bacterial culture alone (Table 4), which was used as the gold-standard comparator method, were 99.1% and 91.8%, respectively. In addition, the PPV and NPV of the Xpert GBS LB XC test were 71.6% and 99.8%, respectively, compared to the bacterial culture method alone, which had a 17.2% GBS positivity rate (Table 4).

**TABLE 4:** Performance of the Xpert GBS LB XC test versus enriched bacterial culture

Xpert GBS LB XC result & test performance	Enriched bacterial culture results		
	Positive	Negative	Total
Positive	106	42	148
Negative	1	472	473
Total	107	514	621
Sensitivity	99.1%	95% CI^*a*^ = 94.9%−99.8%	
Specificity	91.8%	95% CI = 89.1%−93.9%	
Positive predictive value	71.6%	95% CI = 63.9%−78.3%	
Negative predictive value	99.8%	95% CI = 98.8%−100.0%	
Positivity rate	17.2%	95% CI = 14.5%−20.4%	

a CI, confidence interval.

## DISCUSSION

Since the CDC recommended culture-based GBS screening to assess antepartum GBS colonization in 2010, several commercial assays utilizing nucleic acid amplification techniques have been cleared by FDA. In 2019, the most recent laboratory testing guidelines curated by ASM’s Subcommittee on Laboratory Practices for prenatal GBS screening described both NAAT and culture-based screening of GBS from the enriched broth as acceptable options for assessing the GBS colonization status of prenatal women (5). Laboratories that are still performing culture-based screening must identify both hemolytic and nonhemolytic GBS colonies by selective agar medium and/or CAMP test, latex agglutination, MALDI-TOF MS, and other appropriate tests (5). Many laboratories have replaced conventional culture-based identification with NAAT platforms for rapid turnaround time and enhanced sensitivity and specificity. Most commercial molecular GBS assays target the various regions of the *cfb* gene, while very few assays target a combination of *cfb* and other genes or completely different genomic regions of interest to identify GBS from rectovaginal samples enriched in the broth medium (9).

Upon investigating discrepant results for the Xpert GBS LB XC test versus the composite comparator method comparison, six samples were positive using the Xpert GBS LB XC test but negative using the composite comparator method. This means that those six samples were negative using both the Fusion GBS test and bacterial culture. The most plausible explanation for this discrepancy is low bacterial load even after nutrient enrichment. Although reviewing the threshold cycle (*C_T_*) values from the Xpert GBS LB XC test could suggest that low target levels likely contributed to the discrepancy, the *C_T_* values are not part of product labeling. Exposure of bacteria to antibiotics prior to sample collection could be one of the explanations for negative bacterial culture results. Processing samples on the Fusion GBS assay requires adding nutrient broth to the sample lysis tube, which could contribute to the additional dilution of the samples containing low levels of GBS, which then may be below the threshold of the Fusion GBS assay. Moreover, it is impossible to determine if these discrepancies were due to differences in the threshold without serotype identification. The limit of detection (LoD) of the Fusion GBS assay ranges from 84 to 301 CFU/mL, whereas that of the Xpert GBS LB XC ranges from 40 to 682 CFU/mL. Additionally, testing all samples at the same time/day on each platform was not possible logistically. One sample was negative according to the Xpert GBS LB XC test but positive using the composite comparator method. Specifically, it was positive only by bacterial culture but negative using the Fusion GBS assay (i.e., Xpert negative, Panther negative, culture positive). There was no obvious root cause identified for this discordant result.

For investigation of the discrepant results for the Xpert GBS LB XC test versus the enriched bacterial culture comparison, 42 samples were positive using the Xpert GBS LB XC test but negative by enriched bacterial culture. Of these, 36 were positive using the Fusion GBS assay, in agreement with the Xpert GBS LB XC test, and 6 were negative using the Fusion GBS assay. One sample was negative using the Xpert GBS LB XC but positive using enriched bacterial culture; it was also negative using the Fusion GBS assay. No obvious issue or root cause was identified for these 43 (42 + 1) samples, with discrepant results between the Xpert GBS LB XC and enriched bacterial culture. However, the discrepant results were reviewed per study protocol. The 36 discrepant samples that were negative by culture but positive by both NAAT methods could likely be due to sample instability. Twenty-eight percent (42/148) of potentially critical cases were missed by the traditional bacterial culture method in this study. The percent positivity based on culture only would be 17.2%, as opposed to 23.0% with the composite comparator method, indicating the higher sensitivity and reliability of NAAT. This finding is consistent with the report from Buchan et al. (3).

The strength of this study was its large sample size across three different geographical regions in the United States, reflecting the diverse patient population and possibly encompassing mutant strains that may have otherwise been undetected by the *cfb* gene targeted assay. One major limitation was the study’s inability to determine how many *cfb* mutant GBS strains were captured by the new Xpert GBS LB XC test that the Xpert GBS LB test could have otherwise missed; this is due to the nature of a controlled, blinded study, where discrete users performed testing of Xpert and the comparator method. This could have strengthened the data, as the clinical samples tested represented patient populations from three distinct geographical regions. This also could have been potentially addressed by subculturing the isolates into Lim broth and testing them with the Xpert GBS LB test or by parallel testing of the Lim broth samples with both the Xpert GBS LB XC and Xpert GBS LB tests. As per Tickler et al., *cfb* mutant GBS strains are very rare in the United States, at <1% in multiple geographic regions (8). Therefore, approximately 1 isolate in this study would be predicted to have carried the *cfb* mutation and be captured by Xpert GBS LB XC but missed by the Xpert GBS LB test. From the scientific and epidemiologic standpoints, monitoring the prevalence of both genetically and phenotypically evolved strains from clinical samples over time is crucial and may ultimately impact the need for modified molecular assays and revised laboratory diagnostic protocols.

The finding of mutant GBS strains harboring four different chromosomal deletions in or near the region that encodes *cfb* that may escape detection by molecular assays targeting *cfb* (8) raises valid clinical concerns. Similarly, the deletion of a large portion of the *cfb* gene (10) or the entire *cfb* gene (11) may even cause false-negative CAMP test results. Defective transcription of the *cfb* gene may also cause CAMP negative results (11, 12). Laboratories that are still relying on the CAMP test without additional testing to confirm GBS are likely bound to a similar pitfall as the molecular tests targeting the *cfb* gene alone.

Surveillance of the clinical samples from four distinct geographic regions of the United States showed a local cluster of clonal deletion in one U.S. state (8). However, this occurrence is not limited to the United States, based on sporadic case reports, including those from China (10), Italy (13), and one chromosomally deleted strain from Ireland identified by Tickler et al. (8). Interestingly, defective or mutant *cfb* strains associated with negative CAMP reactions were observed in the veterinary setting of GBS isolated from bovine mastitis (6, 12), prior to the report of human clinical isolates. No known connection has yet been made regarding genetic diversity between veterinary sources and human GBS strains.

The perceived notion of the presence of the *cfb* gene in every GBS strain is no longer applicable with the emergence of mutant strains due to chromosomal recombination events resulting from the selective pressure exerted by the organism. Therefore, continuous surveillance is imperative for identifying the prevalence of mutant strains and potentially evasive clinical isolates. Although the occurrence of mutant GBS strains with a deletion in the *cfb* gene appears low, diagnostic test manufacturers remain vigilant, as these are continuously evolving organisms, and it is thus necessary to ensure that optimal diagnostic accuracy is achieved. Considering a multitarget assay approach for the next generation of molecular GBS tests may address potential diagnostic-evasive strains.

Notably, the performance of the Xpert GBS LB XC test is comparable to that of the composite NAAT-culture method. With the ease of use and reliable performance of the Xpert test in lieu of traditional cultures, it raises the question of whether the conventional culture-based screening method should still be considered a gold standard. Nonetheless, introducing the new, modified Xpert GBS LB XC test with dual gene targets would accurately capture GBS colonization in antepartum women.
